# Baicalin alleviates angiotensin II‐induced cardiomyocyte apoptosis and autophagy and modulates the AMPK/mTOR pathway

**DOI:** 10.1111/jcmm.18321

**Published:** 2024-05-07

**Authors:** Ying Cheng, Mengchao Yan, Shuyu He, Yi Xie, Lihui Wei, Bihan Xuan, Zucheng Shang, Meizhu Wu, Huifang Zheng, Youqin Chen, Meng Yuan, Jun Peng, Aling Shen

**Affiliations:** ^1^ Academy of Integrative Medicine Fujian University of Traditional Chinese Medicine Fuzhou Fujian China; ^2^ Fujian Key Laboratory of Integrative Medicine on Geriatrics Fujian University of Traditional Chinese Medicine Fuzhou Fujian China; ^3^ Fujian Collaborative Innovation Center for Integrative Medicine in Prevention and Treatment of Major Chronic Cardiovascular Diseases Fuzhou Fujian China; ^4^ Innovation and Transformation Center Fujian University of Traditional Chinese Medicine Fuzhou Fujian China; ^5^ Department of Pediatrics Rainbow Babies and Children's Hospital and Case Western Reserve University School of Medicine Cleveland Ohio USA

**Keywords:** Baicalin, angiotensin II, apoptosis, autophagy, AMPK/mTOR pathway

## Abstract

As a main extraction compound from *Scutellaria baicalensis* Georgi, Baicalin exhibits various biological activities. However, the underlying mechanism of Baicalin on hypertension‐induced heart injury remains unclear. In vivo, mice were infused with angiotensin II (Ang II; 500 ng/kg/min) or saline using osmotic pumps, followed by intragastrically administrated with Baicalin (5 mg/kg/day) for 4 weeks. In vitro, H9C2 cells were stimulated with Ang II (1 μM) and treated with Baicalin (12.5, 25 and 50 μM). Baicalin treatment significantly attenuated the decrease in left ventricular ejection fraction and left ventricular fractional shortening, increase in left ventricular mass, left ventricular systolic volume and left ventricular diastolic volume of Ang II infused mice. Moreover, Baicalin treatment reversed 314 differentially expressed transcripts in the cardiac tissues of Ang II infused mice, and enriched multiple enriched signalling pathways (including apoptosis, autophagy, AMPK/mTOR signalling pathway). Consistently, Baicalin treatment significantly alleviated Ang II‐induced cell apoptosis in vivo and in vitro. Baicalin treatment reversed the up‐regulation of Bax, cleaved‐caspase 3, cleaved‐caspase 9, and the down‐regulation of Bcl‐2. Meanwhile, Baicalin treatment alleviated Ang II‐induced increase of autophagosomes, restored autophagic flux, and down‐regulated LC3II, Beclin 1, as well as up‐regulated SQSTM1/p62 expression. Furthermore, autophagy inhibitor 3‐methyladenine treatment alleviated the increase of autophagosomes and the up‐regulation of Beclin 1, LC3II, Bax, cleaved‐caspase 3, cleaved‐caspase 9, down‐regulation of SQSTM1/p62 and Bcl‐2 expression after Ang II treated, which similar to co‐treatment with Baicalin. Baicalin treatment reduced the ratio of p‐AMPK/AMPK, while increased the ratio of p‐mTOR/mTOR. Baicalin alleviated Ang II‐induced cardiomyocyte apoptosis and autophagy, which might be related to the inhibition of the AMPK/mTOR pathway.

## 1 INTRODUCTION

Hypertensive heart disease (HHD) is a significant and common type of cardiovascular disease associated with relatively high morbidity and mortality worldwide.^1^ As the most important risk factors for development of HHD, long‐term uncontrolled hypertension can lead to changes in the cardiac structure and function among patients with HHD, subsequently resulting in heart failure and an increased risk of sudden death.^2^, ^3^ Furthermore, chronically elevated blood pressure leads to increase of cardiac overload, left ventricular systolic dysfunction and eventually death due to heart failure.^3^, ^4^ In general, the development of HHD usually involves in alterations of cardiac structure, shape, function, tissue characterization and scar quantification.^5^, ^6^ Apoptosis, a type of programmed cell death triggered by irreversible DNA damage, is characterized by biochemical and morphological changes.^7^ Of particular significance, cardiomyocyte apoptosis plays a crucial role in various physiological processes throughout the progression of systolic cardiac dysfunction and heart failure.^8^, ^9^, ^10^ Thus, mitigating cardiomyocyte apoptosis constitutes an effective strategy for enhancing cardiac function and improving the quality of life of individuals with hypertension.

Autophagy is a tightly regulated process that recycles misfolded proteins and damaged organelles to maintain normal cellular homeostasis.^11^ Apart from this, autophagy also contributes to the maintenance of cardiac structure and function under physiological conditions.^12^ However, autophagy over‐activation causes excessive protein degradation and cell death in response to pathological conditions.^13^ As is well known, the factors that induce autophagy over‐activation include ischemia/reperfusion injury, oxidative stress, endoplasmic reticulum stress and mitochondrial damage.^14^ Many evidences indicated that the cardiac autophagy was closely related to the pathogenesis of apoptosis and speed up cell death of cardiomyocytes.^15^, ^16^ The inhibition of excessive autophagy could alleviate the development of many heart diseases, such as pressure overload, ischemia/reperfusion injury, cardiac remodelling, and heart failure.^17^, ^18^, ^19^ Autophagy over‐activation leads to programmed cell death, aggravates cardiac remodelling and stimulates heart failure.^20^, ^21^ Thus, it was speculated that autophagy inhibition might protect cardiac apoptosis.

AMP‐dependent protein kinase/mammalian target of rapamycin (AMPK/mTOR) signalling pathway are involved in hypertension‐induced autophagy.^22^ AMPK, an intracellular energy response regulator, could directly/indirectly inhibit mTOR.^23^, ^24^ Such signalling regulates various target genes, including LC3B, Beclin1 and SQSTM1/p62, and ultimately promotes autophagy.^25^, ^26^ As reported that angiotensin II (Ang II)‐induced cardiac dysfunction might be associated with excessively maladaptive autophagy by activating AMPK/mTOR signalling pathway.^27^ Therefore, suppressing the AMPK/mTOR signalling pathway may mitigate hypertension‐induced cardiomyocyte autophagy.

*Scutellaria baicalensis* Georgi (*S. baicalensis*), also known as huang‐qin, is a widely cultivated medicinal plant species in China, with baicalin, baicalein, wogonin, wogonoside, norwogonin and norwogonoside being the most abundant flavones present in its roots.^28^ Among that, Baicalin (5,6‐dihydroxyflavone‐7‐O‐D‐glucuronic; C21H18O11; CAS No. 21967–41‐9) has been certified to be effective in anti‐oxidant, anti‐inflammatory, anti‐fibrotic, anti‐apoptotic and suppressing or inducing autophagy activity.^29^, ^30^, ^31^, ^32^, ^33^ Actually, studies have found that Baicalin could stabilize blood pressure and prevent early heart damage.^34^, ^35^ Evidence also revealed the protective effects of Baicalin on cardiac hypertrophy in spontaneous and renal hypertension rats.^36^ It reported that Baicalin could suppress excessive autophagy, thereby attenuating hyperglycemia‐induced embryonic cardiovascular malformation.^37^ However, the underlying mechanisms of Baicalin on Ang II‐induced cardiomyocyte autophagy and apoptosis remain to be further addressed. Therefore, we speculate that Baicalin might alleviate Ang II‐induced cardiomyocyte apoptosis and autophagy by mediating AMPK/mTOR signalling pathway based on RNA sequencing analysis. To verify this hypothesis, Ang II‐induced C57BL/6 mice in vivo and H9C2 cells in vitro injury model were established to investigate whether Baicalin treatment could attenuate cardiac dysfunction, thereby providing new insights for the molecular mechanism of Baicalin.

## 2 MATERIALS AND METHODS

### 2.1 Materials

Baicalin was provided by Sigma‐Aldrich (purity >95%; Darmstadt, Germany). ALZET® osmotic pump was purchased from Durect Corporation (Cupertino, CA, USA). Dulbecco's Modified Eagle Medium (DMEM), fetal bovine serum (FBS), trypsin–EDTA, trypsin, bicinchoninic acid (BCA) protein assay reagent kit and western blot stripping buffer were obtained from Thermo Fisher Scientific (Waltham, MA, USA). TUNEL Apoptosis Detection Kit I POD was provided by Boster Biological Technology (Wuhan, Hubei, China). Annexin V‐AbFluor™ 647 Apoptosis Detection kit (Red fluorescence) was obtained from Abbkine Scientific (Wuhan, Hubei, China). Antigen repair solution, UltraSensitive™ SP (Mouse/Rabbit) immunohistochemistry (IHC) kit and Diaminobenzidine (DAB) kit were purchased from Maixin Biotechnology (Fuzhou, Fujian, China). Monodansylcadaverine (MDC) kits were provided by Solarbio Science & Technology Co., Ltd. (Beijing, China). Western & IP cell lysis buffer and enhanced chemiluminescence (ECL) kit were purchased from Beyotime Biotechnology (Shanghai, China). 3‐methyladenine (3‐MA) was purchased from MedChemExpress (Monmouth Junction, NJ, USA).

Ang II (Cat no. ab120183) and antibody against LC3B (Cat no. ab48394), SQSTM1/p62 (Cat no. ab91526), Beclin 1 (Cat no. ab62557), AMPK (Cat no. ab32047), p‐mTOR (Cat no. 109268) were purchased from Abcam (Cambridge Science Park, Cambridge, UK). Antibodies against p‐AMPK (Cat no. 2535S), mTOR (Cat no. 2983S), Bax (Cat no. 2772S), cleaved‐caspase 3 (Cat no. 9662S), cleaved‐caspase 9 (Cat no.9508S), anti‐rabbit secondary antibody (Cat no. 7074) and anti‐mouse secondary antibody (Cat no. 7076) were purchased from Cell Signalling Technology (Beverly, MA, USA). Antibodies against Bcl‐2 (Cat no. ABM0010) and GAPDH (Cat no. ABL1021) were obtained from Abbkine Scientific (Wuhan, Hubei, China).

### 2.2 Animals and experimental protocols

Male C57BL/6 (8 weeks aged) was purchased from SLAC Laboratory Animal Technology Co., Ltd (Shanghai, China) and housed in a specific pathogen free environment with a constant temperature of 23 ± 1°C and 12 h light/dark cycles. After being acclimated for 1 week, mice were randomly divided into four groups: Control, Ang II and Ang II + Baicalin (*n* = 5 for each group). Mice in Ang II and Ang II + Baicalin groups were infused with Ang II (500 ng/kg/min) via osmotic pumps, while the mice in Control group were received continuous infusion of same amount of normal saline for 4 weeks. Mice were intragastrically administrated with Baicalin (5 mg/kg/day) in Ang II + Baicalin group, while mice in both Control and Ang II groups were intragastrically administrated with an equal volume of double distilled water. All experiments were strictly carried out, and also approved by the Institutional Animal Care and Use Committee of Fujian University of Traditional Chinese Medicine (Approval no. FJTCM IACUC 2020028).

### 2.3 Echocardiographic measurements

At the end of the experiment, transthoracic echocardiography was measured using the Vevo2100 High‐Resolution Imaging System (Visual Sonics, Toronto, ON, Canada). Briefly, mice were anaesthetised with 2% isoflurane and maintained with 1.5% isoflurane. Afterword, they were fixed on the console. The 30‐MHz probe was slowly fine‐tuned in B‐mode to obtain clear cardiac images to the maximum extent possible; 2D left ventricular images were then obtained from M‐mode echocardiograms as described previously.^38^ Left ventricular ejection fraction (LVEF), left ventricular fractional shortening (LVFS), left ventricular mass (LVM), left ventricular diastolic volume (LV Vol;d) and left ventricular systolic volume (LV Vol;s) were calculated by using the Vevo software (Visual Sonics; version: 3.1.0.13029). At least three cardiac cycles were measured for each mouse, and the average value was calculated.

### 2.4 RNA sequencing (RNA‐seq)

The cardiac tissues were stored in the RNAlater solution for RNA‐seq analysis. Total RNA was extracted using TRIzol reagent according to the manufacturer's protocol. The total RNA quality was measured with Qubit 3.0 and Agilent 2100 Bioanalyze (Thermo Fisher Scientific). Total RNA was used for the study when the ratio of RNA integrity was ≥7, ratio of 28S ribosomal RNA (rRNA) to 18S rRNA was ≥1.5:1, and starting requirement range was 0.1–1 μg. RNA‐seq was conducted by the CapitalBio Technology (Beijing, China), as described previously.^39^ The differentially expressed transcripts (DETs) were further compared among different groups (fold‐change ≥2, *p* < 0.05). Moreover, Gene Ontology (GO) and Kyoto Encyclopedia of Genes and Genomes (KEGG) analyses were performed to identify enriched signalling pathways and biological functions.

### 2.5 Terminal deoxynucleotidyl transferase dUTP nick end labeling (TUNEL) staining

According to instructions, cardiomyocyte apoptosis was assessed in the heart sections by TUNEL kit. The slices were initially fixed in 3% H_2_O_2_, followed by treatment with proteinase K for 20 min. Subsequently, they were incubated with the TdT enzyme and biotinylated dUTP, and finally blocked with 50 μL of blocking solution for 30 min. Additionally, a 50 μL of biotinylated anti‐digoxin antibody was added and incubated for 30 min. Subsequently, the streptavidin‐biotin complex was treated for another 30 min. Finally, DAB staining was performed to visualize the results. Photographs were obtained at 400× magnification using an optical microscope (Leica DM6000B, Leica Microsystems, Wetzlar, Germany).

### 2.6 IHC analysis

Cardiac tissues were fixed in 4% paraformaldehyde, embedded in paraffin, and sectioned at a thickness of 4 μM according to standard procedures. After graded ethanol dewaxing, slices were boiled in 10 mM sodium citrate buffer at 100°C for 10 min for antigen retrieval. Following that, they were incubated in 0.25% Triton X‐100 for 10 min, and then blocked with 3% H_2_O_2_ for 10 min. After rinsing in PBS solution for 15 min, slices were incubated with a blocking buffer for 1 h. Each slide was incubated with an appropriate primary antibody (LC3B, SQSTM1/p62, Beclin 1, p‐AMPK, AMPK, p‐mTOR diluted 1:100, mTOR diluted 1:200) at 4°C overnight. Subsequently, the corresponding secondary antibody was incubated for 1 h and treated with a streptavidin‐HRP conjugate 1 h at room temperature. Moreover, the slices were stained with DAB kit and Haematoxylin. Brown‐stained cells were considered positive cells. Finally, images were captured at ×400 magnification using an optical microscope (Leica) and measured by Motic med 6.0 software (Motic China Group, Co., Ltd., Xiamen, China).

### 2.7 Cell culture and treatments

H9C2 cells were obtained from the Cell Bank of the Chinese Academy of Sciences (Shanghai, China) and cultured in DMEM with 10% FBS and 1% penicillin/streptomycin (100 IU/mL; 100 μg/mL). Cells were incubated in a humidified incubator at 37°C with 5% CO_2_. When it reached 80% confluency, H9C2 cells were trypsinized and passaged for subsequent experiments. H9C2 seeded in six‐well plates at a density of 0.6 × 10^5^ cells/well. After 24 h of incubation, H9C2 cells were stimulated with Ang II (1 μM) or treated with Ang II and Baicalin (12.5 μM, 25 μM or 50 μM) for 48 h in DMEM containing 2% FBS.

### 2.8 Flow cytometric analysis

Apoptosis was detected by flow cytometry using Annexin V/PI staining assay according to instructions. After treatment, cells were collected with trypsin (without EDTA), centrifugated (2000 rpm, 3 min), and washed with ice‐cold PBS. Next, 100 μL Annexin V Binding Buffer, 5 μL Annexin V‐APC and 2 μL PI were added and mixed gently. The samples were then incubated at room temperature under dark for 15 min. Finally, apoptosis was detected with a CytoFLEX (Beckman Coulter, Inc., Brea, CA, USA) and analysed by CytExpert software (Beckman Coulter, Inc., Brea, CA, USA).

### 2.9 Western blotting analysis

Tissues and cells were harvested and lysed with ice‐cold lysis buffer containing protease inhibitors on ice for 20 min at 4°C. Following centrifugation at 14,000 rpm for 20 min at 4°C, total protein concentration was quantified using BCA protein assay kit and denatured with 5 × SDS loading buffer for 5 min. Subsequently, 40 μg of equivalent protein in each group was separated into 10% or 12% gradient SDS‐PAGE. Then proteins were transferred from polyacrylamide gels to membranes. The membranes were blocked with 5% skimmed milk for 2 h at room temperature. Membranes were cut horizontally and subsequently incubated with the diluted primary antibodies at 4°C overnight. After being washed with TBST thrice, membranes were incubated with secondary antibodies (1:5000 dilution) for 2 h at room temperature. The ECL kit was used to detect protein expression using the Chemi Doc MP Imaging System (Bio‐Rad Laboratories, Inc., Hercules, CA, USA). For proteins with very close/overlapping MW ranges, membranes were washed with TBST to remove the chemiluminescent substrate. Western blot stripping buffer was added and incubate for 15 min at 37°C. Subsequently, membranes were re‐blocked, incubated with primary and secondary antibodies, and detected with ECL. The optical density of each protein was calculated by Image J software (open‐source Java image processing program at https://imagej.nih.gov/ij/). GAPDH was used as internal control.

### 2.10 Monodansylcadaverine (MDC) staining

MDC, a fluorescent compound, was used as a tracer of autophagic vacuoles. After treatment, H9C2 cells were washed twice with washing buffer and incubated with 300 μL MDC solution (50 μM) for 8–10 min. Photographs were captured with a confocal microscope (UltraVIEW® Vox, PerkinElmer, Santa Clara, CA, USA). Images were taken at 200× magnification. Data analysis was performed using Image J software.

### 2.11 mRFP‐GFP‐LC3 assay

To assess autophagy flux, H9C2 cells were seeded on the coverslips and incubated with an mRFP‐GFP‐LC3 lentivirus at 50 MOI for 6 h before receiving further stimulus. Cells were cultured with AngII followed with or without Baicalin treatment for 48 h. The treated cells were observed under a confocal microscope at 561 and 488 nm using a 1000× oil immersion objective (LSM710, CarlZeiss, Oberkohen, Germany). The puncta of each cell were counted, and at least six pictures were taken for each sample. GFP fluorescence is sensitive to the acidic pH environment within the lysosome, resulting in loss of fluorescence upon fusion of autophagosomes with lysosomes to form autolysosomes. Conversely, RFP fluorescence remains stable under low pH conditions, allowing autolysosomes to exhibit red fluorescence exclusively. Autophagy flux was then measured by confocal counting of GFP^+^/mRFP^+^ (yellow dots, autophagosome) and GFP^−^/mRFP^+^ (red dots, autolysosome) puncta.

### 2.12 Statistical analysis

The statistical significance was assessed using SPSS 23 software. One‐way analysis of variance (ANOVA) was used to compare differences among three or more groups when conforming to a normal distribution. The nonparametric Kruskal–Wallis test was used to compare differences among the three groups when inconsistent with the normal distribution. All data were expressed as the mean ± standard deviation, and *p* < 0.05 was recognized as statistically significant.

## 3 RESULTS

### 3.1 Baicalin treatment ameliorated Ang II‐induced changes in cardiac function

**FIGURE 1:**
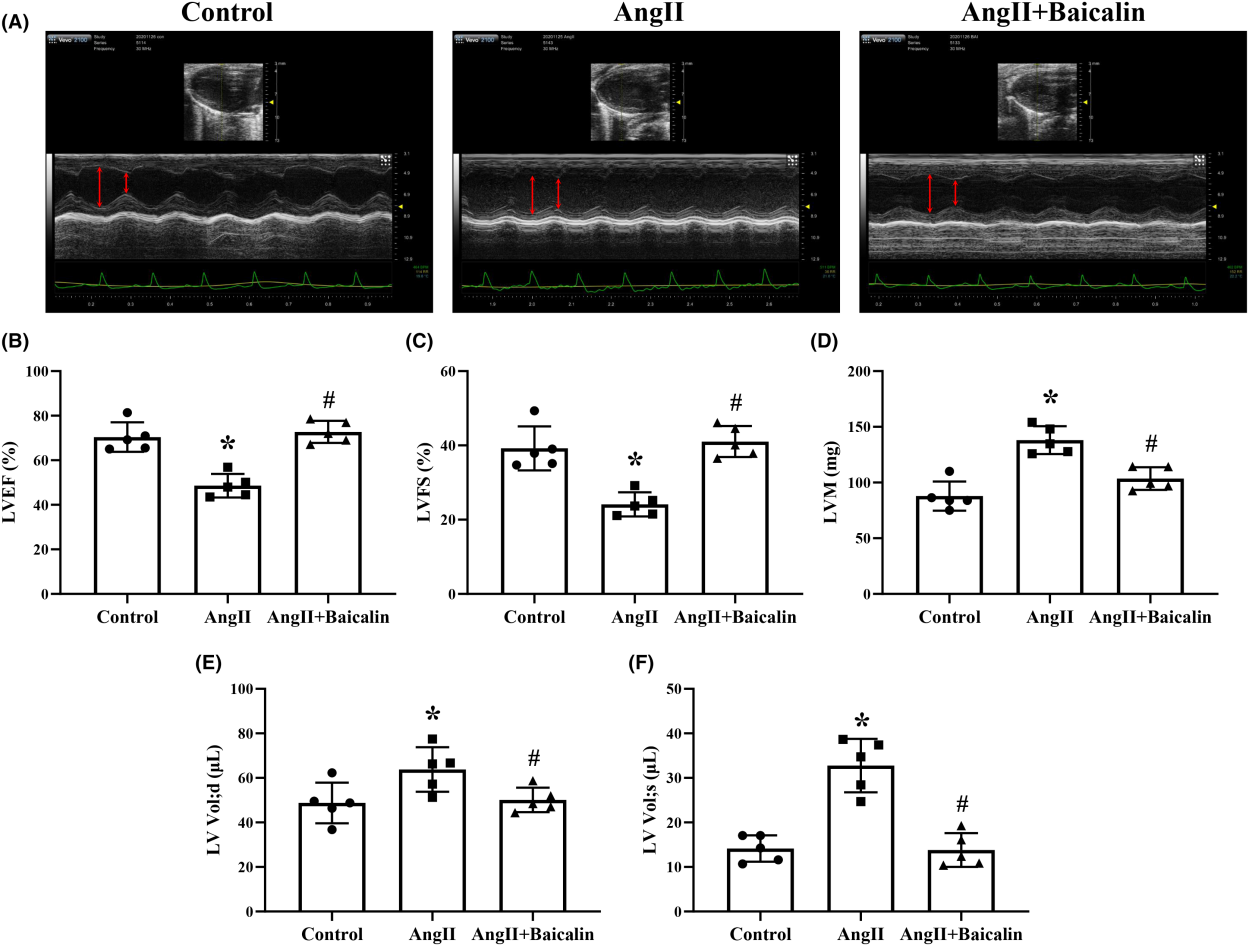
Baicalin treatment ameliorates angiotensin II (Ang II) induced changes in cardiac function. (A) Representative images of echocardiography from each group of mice. (B) Left ventricular ejection fraction (LVEF). (C) Left ventricular fractional shortening (LVFS). (D) Left ventricular mass (LVM). (E) Left ventricular diastolic volume (LV Vol;d). (F) Left ventricular systolic volume (LV Vol;s). *p < 0.05, versus Control group, ^#^ *p* < 0.05, versus Ang II group. All values are mean ± SD (*n* = 5 samples from each group).

Previous study demonstrated that Baicalin treatment attenuated Ang II‐induced elevation of blood pressure (DOI: 10.1016/j.biopha.2021.112124).^40^ In the current study, a cardiac functional study performed using echocardiography revealed that mice receiving Ang II infusion exhibited decreased mean values of LVEF and LVFS and increased values of LVM, LV Vol;d and LV Vol;s (Figure 1A–F) compared to the mice in the Control group, which were all attenuated after Baicalin treatment (Figure 1A–F).

### 3.2 Identification of DETs in cardiac tissues of Ang II‐infused mice following Baicalin treatment

**FIGURE 2:**
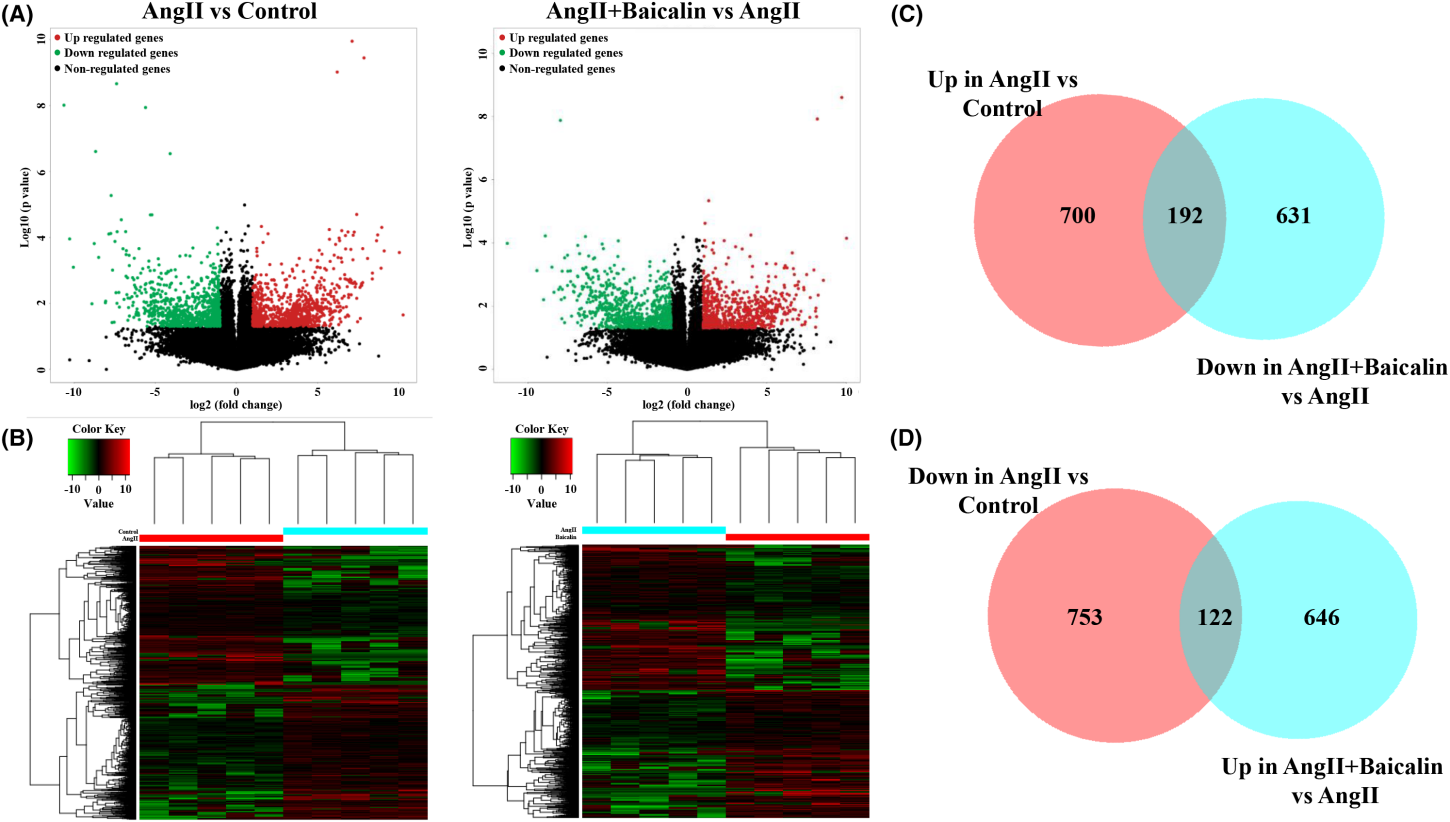
Baicalin treatment on gene expression profiling of cardiac tissues in angiotensin II (Ang II) infused mice. RNA sequencing was performed to determine the differentially expressed transcripts (DETs) in cardiac tissues from each group. (A) Volcano and (B) hierarchical clustering plots were used to compare the gene expression profiles (|fold‐change| ≥2, *p* ≤ 0.05). (C) The overlapping area represents genes that were increased in the Ang II group but were decreased in the Ang II + Baicalin group. (D) The overlapping area represents the genes that were decreased in the Ang II group but were increased in the Ang II + Baicalin group (*n* = 5 samples from each group).

RNA‐seq analysis [NCBI Gene Expression Omnibus accession number: GSE193504] revealed that 892 transcripts were up‐regulated and 875 transcripts were down‐regulated in the cardiac tissues of mice following Ang II infusion (fold‐change ≥2; *p* ≤ 0.05 vs. control; Figure 2A,B). Meanwhile, compared with the Ang II group, 768 transcripts were significantly up‐regulated and 823 transcripts were down‐regulated in the cardiac tissues of mice following Baicalin treatment (fold‐change ≥2; *p* ≤ 0.05 vs. Ang II; Figure 2A,B). Among these DETs, Baicalin treatment significantly reversed 192 up‐regulated and 122 down‐regulated DETs in the Ang II group (Figure 2C,D). In addition, the details of the top 20 DETs are listed in Table 1.

**TABLE 1:** The top 20 differentially expressed genes.

ID	logFC	*p‐*value	Symbol	Description	Regulation
ENSMUST00000133325	11.30	0.00	Got1	Glutamic‐oxaloacetic transaminase 1	Down
ENSMUST00000217593	8.93	0.00	Sltm	SAFB‐like, transcription modulator	Down
ENSMUST00000106858	7.52	0.00	Mier1	Mesoderm induction early response protein 1	Down
ENSMUST00000109159	7.34	0.00	Tshz2	Teashirt zinc finger family member 2	Down
ENSMUST00000107704	6.89	0.00	Msantd3	Myb/SANT‐like DNA‐binding domain‐containing protein 3	Down
ENSMUST00000216841	6.81	0.00	Ubl7	Ubiquitin‐like 7	Down
ENSMUST00000160758	6.76	0.01	Mycbp2	MYC binding protein 2, E3 ubiquitin protein ligase	Down
ENSMUST00000102660	6.71	0.00	Sestd1	SEC14 domain and spectrin repeat‐containing protein 1	Down
ENSMUST00000153488	6.58	0.00	Naa30	N‐alpha‐acetyltransferase 30	Down
ENSMUST00000136663	6.09	0.00	Mink1	Misshapen‐like kinase 1	Down
ENSMUST00000221142	8.57	0.00	Nol8	Nucleolar protein 8	Up
ENSMUST00000114167	7.01	0.00	Camsap1	Calmodulin‐regulated spectrin‐associated protein 1	Up
ENSMUST00000119854	6.56	0.01	Limch1	LIM and calponin homology domains‐containing protein 1	Up
ENSMUST00000015994	6.08	0.02	Adamtsl4	ADAMTS‐like protein 4	Up
ENSMUST00000193404	5.67	0.01	Pcdhga10	Protocadherin gamma subfamily A, 10	Up
ENSMUST00000148750	5.31	0.04	Slc4a4	Solute carrier family 4 (anion exchanger), member 4	Up
ENSMUST00000030164	5.29	0.03	Vcp	Valosin containing protein	Up
ENSMUST00000100750	5.14	0.02	Mecp2	Methyl‐CpG‐binding protein 2	Up
ENSMUST00000086325	5.12	0.00	Flywch1	FLYWCH‐type zinc finger 1	Up
ENSMUST00000209098	5.11	0.02	Nap1l4	Nucleosome assembly protein 1‐like 4	Up

### 3.3 Enrichment of GO based on DETs

**FIGURE 3:**
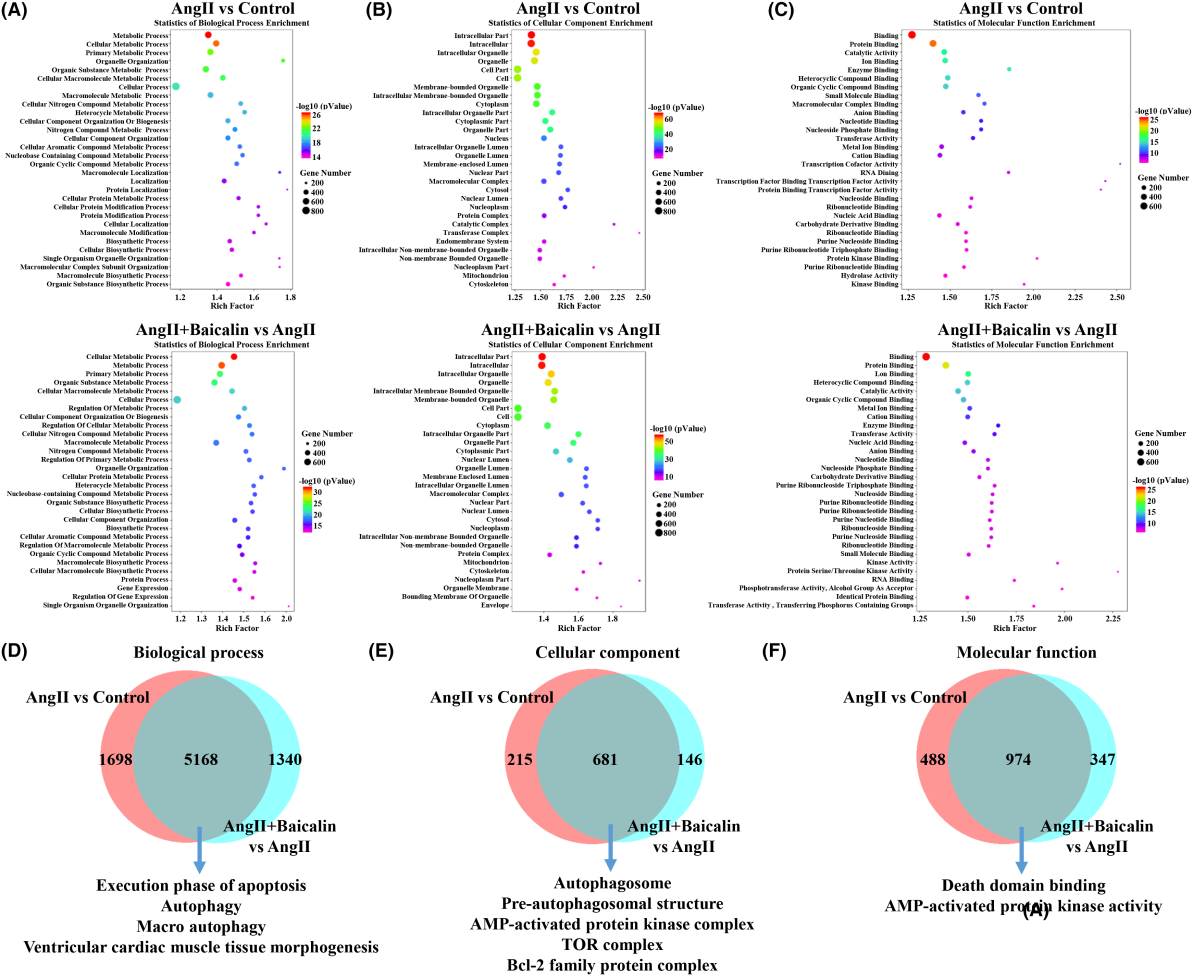
Gene Ontology (GO) enrichment analysis. GO enrichment analysis was performed based on the differentially expressed transcripts (DETs) from both comparisons of Ang II versus Control and Ang II + Baicalin versus Ang II. The top 30 enriched items of biological process (A), cellular component (B) and molecular function (C). The overlapping biological targets between the Ang II versus Control groups and Ang II + Baicalin versus Ang II groups were analysed from three aspects of biological process (D), cellular component (E) and molecular function (F).

The DETs between the Ang II and Control groups were used for the GO enrichment analysis including the biological process (BP), cellular component (CC) and molecular function (MF). As shown in Figure 3A–C, BP analysis demonstrated that potential target genes were associated with metabolic process, cellular metabolic process and primary metabolic process terms. In CC analysis, DETs were primarily associated with intracellular part, intracellular and intracellular organelle terms. MF analysis revealed that potential target genes were associated with binding, protein binding and catalytic activity terms. Furthermore, the GO enrichment analysis of DETs observed on comparing the Ang II + Baicalin and Ang II groups predicted multiple potential functions (Figure 3A–C). In BP analysis, these DETs were significantly associated with cellular metabolic process, metabolic process and primary metabolic process terms. CC analysis revealed that these DETs were associated with intracellular part, intracellular and intracellular organelle terms. MF analysis revealed that these DETs were associated with binding, protein binding and ion binding terms. Moreover, on comparing the Ang II and Control groups and Ang II + Baicalin and Ang II groups, the present study identified 5168 common enriched BP terms (Figure 3D), 681 common enriched CC terms (Figure 3E) and 974 common enriched MF terms (Figure 3F). Among them, the regulation of execution phase of apoptosis, autophagy, macro autophagy and ventricular cardiac muscle tissue morphogenesis of BP, the regulation of autophagosome, pre‐autophagosomal structure, AMP‐activated protein kinase complex and TOR complex and Bcl‐2 family protein complex of CC, the regulation of death domain binding and AMP‐activated protein kinase activity of MF were significantly enriched.

### 3.4 Enrichment of KEGG pathways based on DETs

**FIGURE 4:**
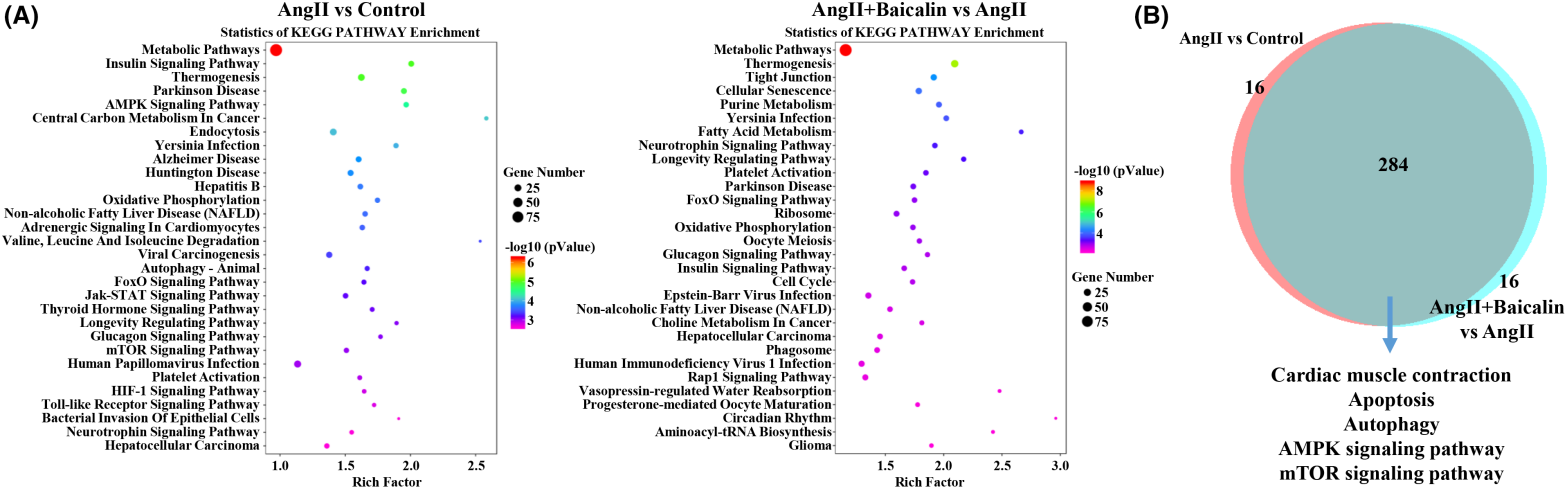
Kyoto Encyclopedia of Genes and Genomes (KEGG) pathway enrichment analysis. (A) KEGG enrichment analysis was performed to identify the enriched signalling pathway in both comparisons of the Ang II versus Control groups and Ang II + Baicalin versus Ang II groups. The top 30 enriched signalling pathways are presented. (B) The overlapped area represents the number of enriched signalling pathways in both the comparisons of Ang II versus Control groups and Ang II + Baicalin versus Ang II groups.

KEGG analysis pathway enrichment analysis enriched multiple signalling pathways and top 30 most significantly enriched pathways were presented in Figure 4A, including Metabolic pathways, Insulin signalling pathway, Thermogenesis, Parkinson disease and AMPK signalling pathway based on the DETs between Ang II and Control groups. On the other hand, Metabolic pathways, Thermogenesis, Tight junction, Cellular senescence and Purine metabolism were significantly enriched based on the DETs between Ang II + Baicalin and Ang II groups. In addition, an intersection analysis identified 284 overlapping pathways (Figure 4B), including cardiac muscle contraction, apoptosis, autophagy, AMPK signalling pathway and mTOR signalling pathway. Both GO and KEGG enrichment analyses indicated that apoptosis, autophagy and AMPK/mTOR signalling pathway might be the potential regulatory mechanisms of Baicalin treatment against HHD.

### 3.5 Baicalin treatment attenuated Ang II‐induced cardiomyocyte apoptosis in vivo and in vitro

**FIGURE 5:**
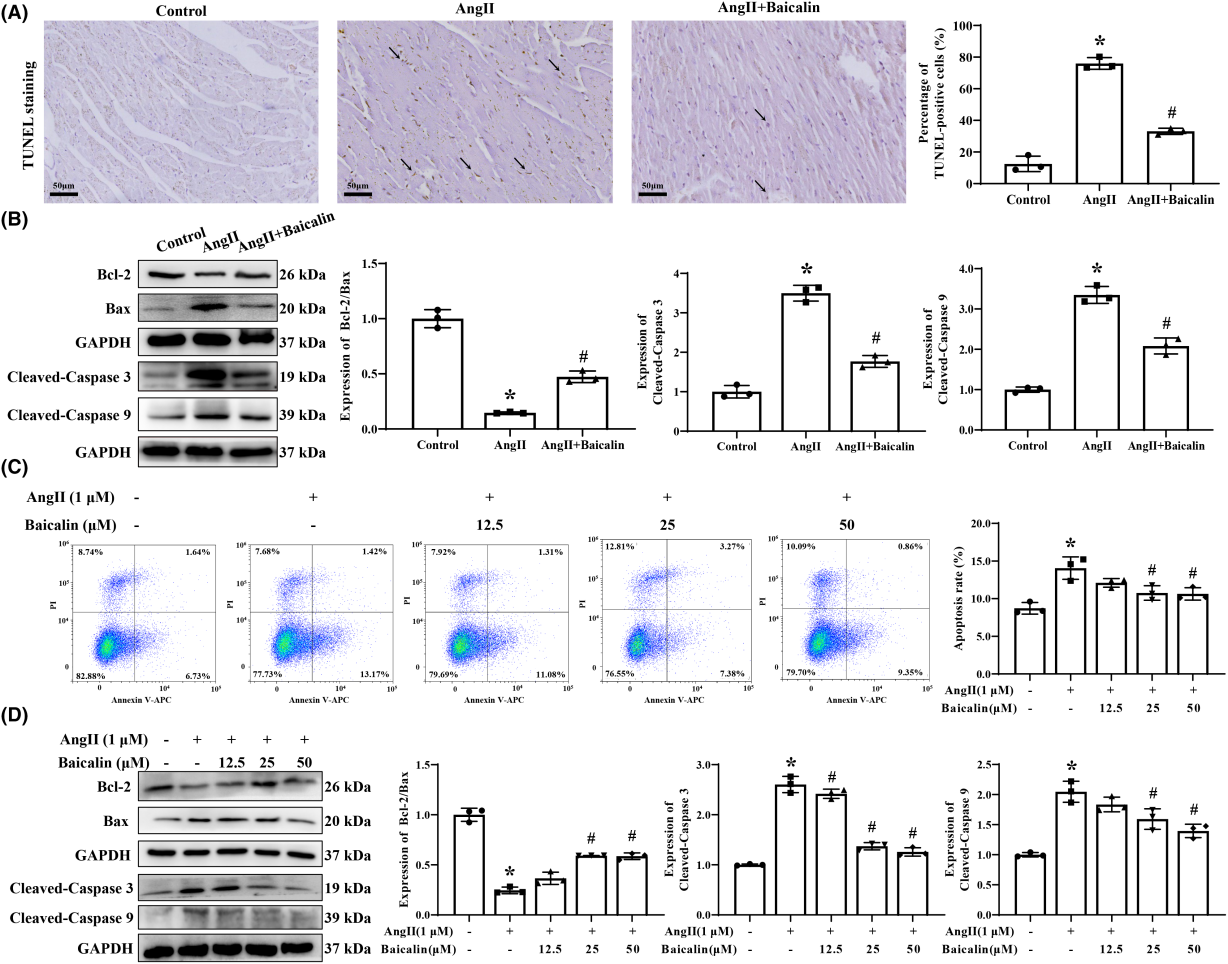
The effects of Baicalin on angiotensin II (Ang II) infused cardiomyocyte apoptosis in vivo and in vitro. (A) The representative images of terminal deoxynucleotidyl transferase dUTP nick end labeling (TUNEL) staining from each group of mice. Images were magnified at 400×, and the positive rate was calculated. (B) The expression of Bax, Bcl‐2, cleaved‐caspase 3, and cleaved‐caspase 9 was analysed via western blotting in mice heart tissue. (C) Annexin V/PI staining was used to measure cell apoptosis via flow cytometry and analysed via CytExpert software. H9C2 cells were treated with 1 μM Ang II in the presence of 12.5, 25 or 50 μM Baicalin for 48 h. (D) The expression of Bax, Bcl‐2, cleaved‐caspase 3, and cleaved‐caspase 9 was analysed via western blotting in H9C2 cells. GAPDH was included as a loading control. *p < 0.05, versus Control group, ^#^ *p* < 0.05, versus Ang II group. All values are mean ± SD (*n* = 3 samples from each group).

TUNEL staining revealed that Ang II infusion significantly increased the percentage of TUNEL positively stained cells in cardiac tissues of mice, which were significantly abrogated after Baicalin treatment (Figure 5A). Baicalin treatment significantly attenuated the up‐regulation of cleaved‐caspase 3, cleaved‐caspase 9 and Bax, and down‐regulation Bcl‐2 protein levels in cardiac tissues of Ang II infused mice (Figure 5B).

In vitro study, Annexin V/PI staining showed that Ang II simulation increased the percentage of apoptotic H9C2 cells, which were alleviated after different concentrations of Baicalin treatment (Figure 5C). Consistent with in vivo study, Baicalin treatment decreased the expression of cleaved‐caspase 3, cleaved‐caspase 9, and Bax and increased the expression of Bcl‐2 in the Ang II‐induced H9C2 cells (Figure 5D). Therefore, these results suggested that Baicalin treatment ameliorated the Ang II‐induced cardiomyocyte apoptosis both in vivo and in vitro.

### 3.6 Baicalin treatment suppressed cardiomyocyte autophagy in vivo and in vitro

**FIGURE 6:**
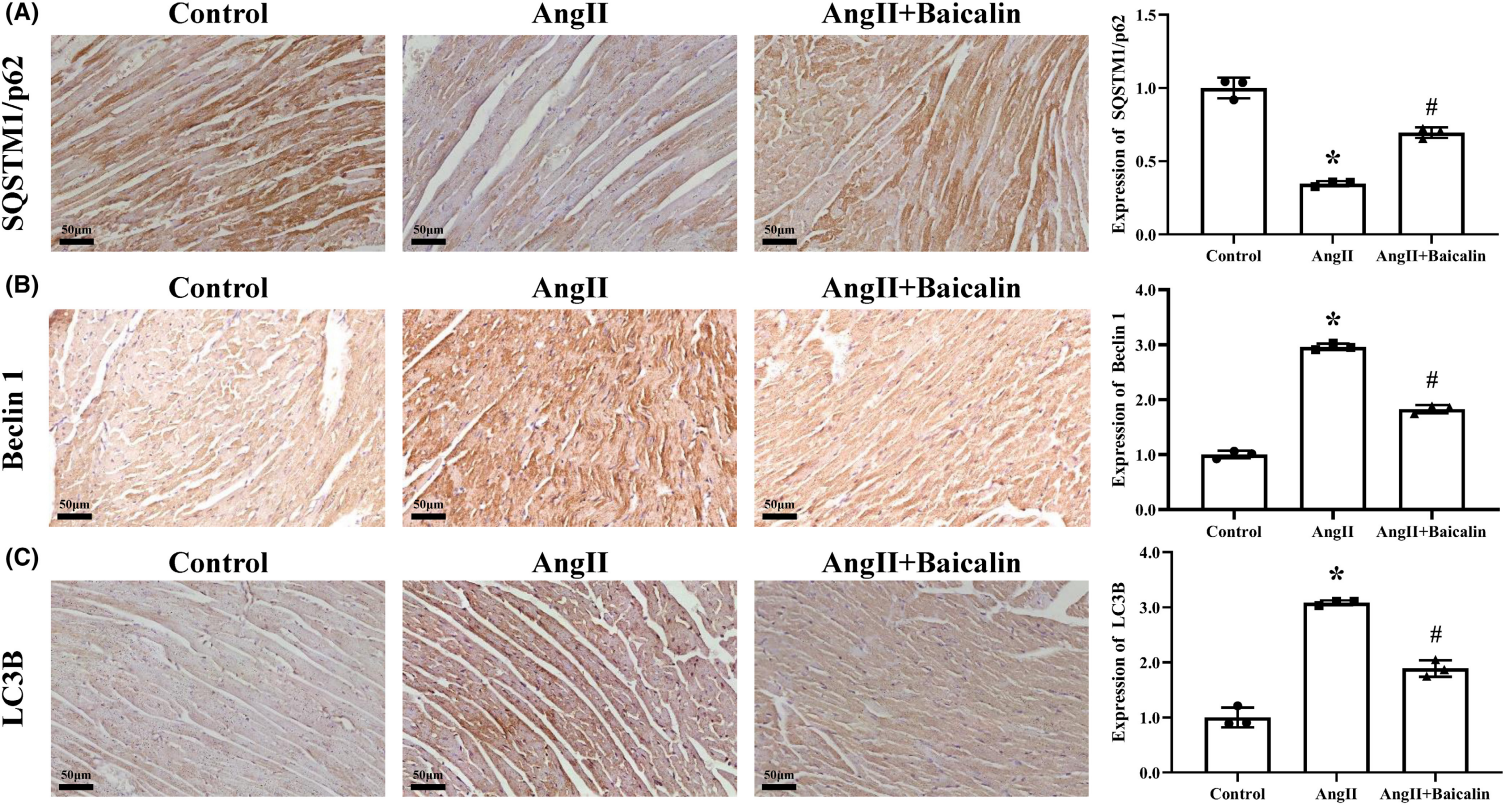
The effects of Baicalin on angiotensin II (Ang II) induced cardiomyocyte autophagy in vivo. Immunohistochemistry was used to detect the expression of (A) SQSTM1/p62, (B) Beclin 1, and (C) LC3B in mice cardiac tissues at a magnification of 400×. *p < 0.05, versus Control group, ^#^ *p* < 0.05, versus Ang II group. All values are mean ± SD (*n* = 3 samples from each group).

IHC analysis showed that Beclin 1 and LC3B expression were up‐regulated, and SQSTM1/p62 was down‐regulated in cardiac tissues of Ang II‐infused mice, which was reversed after Baicalin treatment (Figure 6A–C).

**FIGURE 7:**
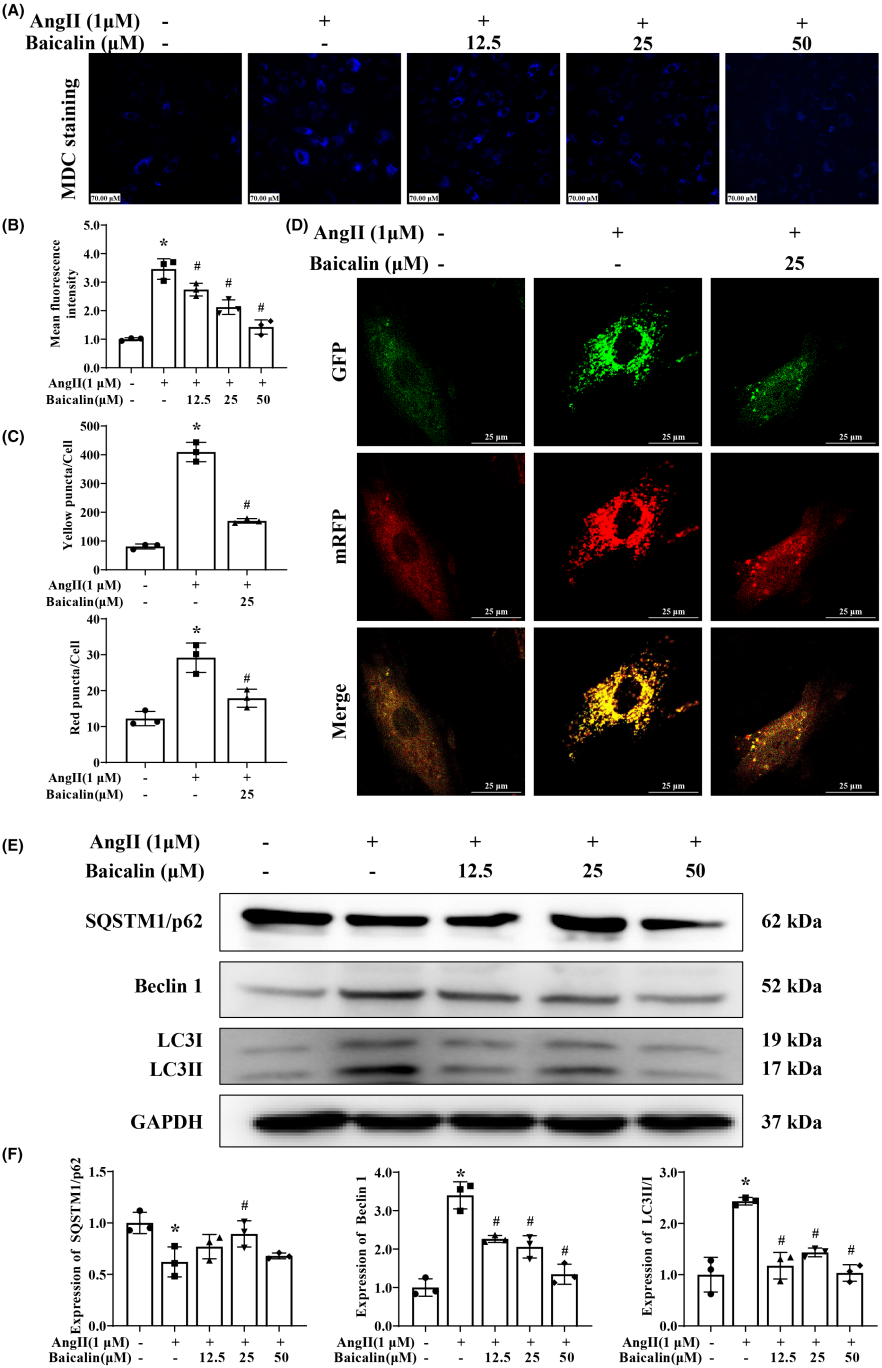
The effects of Baicalin on angiotensin II (Ang II) induced cardiomyocyte autophagy in vitro. (A, B) Representative photomicrographs and quantification of Monodansylcadaverine (MDC) staining at a magnification of 200×. The mean fluorescence intensity of MDC‐stained were quantified. H9C2 cells were treated with Ang II (1 μM) in the presence of 12.5, 25 or 50 μM Baicalin for 48 h. (C, D) The representative photomicrographs of confocal fluorescent images of H9AC2 cells with the tandem fluorescent‐tagged LC3 assay at a magnification of 1000×. Quantification of the number of GFP^+^/mRFP^+^ (yellow dots, autophagosome) and GFP^−^/mRFP^+^ (red dots, autolysosome) puncta in H9C2 cells. (E, F) Western blotting was used to detect the expression of SQSTM1/p62, Beclin 1 and LC3II in the H9C2 cells. GAPDH was included as a loading control. *p < 0.05, versus Control group, ^#^ *p* < 0.05, versus Ang II group. All values are mean ± SD (*n* = 3 samples from each group).

Meanwhile, MDC staining observed an increase of autophagosomes with blue granular structures in Ang II stimulated H9C2 cells, which were decreased after various concentrations of Baicalin treatment (Figure 7A,B). H9C2 cells were then transfected with adenovirus harbouring tandem fluorescent mRFP‐GFP‐LC3 to elucidate the specific step of autophagic flux affected by Baicalin. Interestingly, Ang II stimulation elevated the basal autophagic level of H9C2 cells as evidence by the increased numbers of both yellow puncta (autophagosome) and red puncta (autolysosome). Besides, Baicalin treatment blocked the effects of Ang II on autophagic flux regulation (Figure 7C,D). Consistent with in vivo study, Baicalin treatment attenuated the up‐regulation of Beclin 1 and LC3II, and the down‐regulation of  SQSTM1/p62 at the proteins level in the Ang II‐induced H9C2 cells (Figure 7E,F).

**FIGURE 8:**
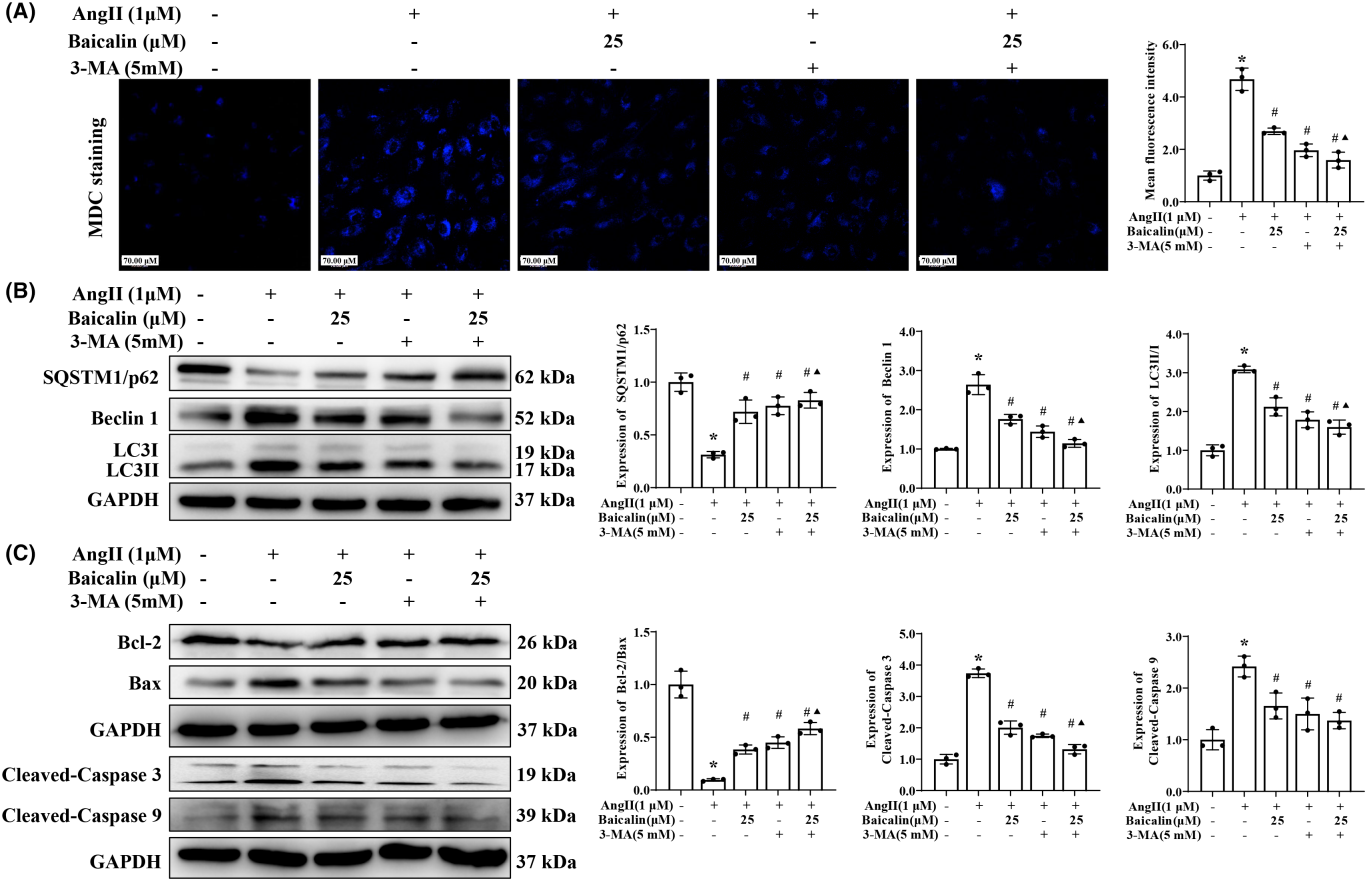
The effects of 3‐methyladenine (3‐MA) and Baicalin on angiotensin II (Ang II) induced cardiomyocyte autophagy in H9C2 cells. (A) Representative photomicrographs and quantification of Monodansylcadaverine (MDC) staining at a magnification of 200×. The mean fluorescence intensity of MDC‐stained were quantified. H9C2 cells were incubated with Ang II (1 μM) for 48 h in the presence or absence of 3‐MA (5 mM) and followed (or not) by Baicalin (25 μM). (B) Western blotting was used to detect the expression of SQSTM1/p62, Beclin 1, and LC3II in the H9C2 cell. GAPDH was included as a loading control. (C) Western blotting was used to detect the expression of Bax, Bcl2, cleaved‐caspase 3, cleaved‐caspase 9. GAPDH was included as a loading control. *p < 0.05, versus Control group, ^#^ *p* < 0.05, versus Ang II group, ^▲^ *p* < 0.05, versus Ang II + Baicalin group. All values are mean ± SD (*n* = 3 samples from each group).

3‐MA, an inhibitor of class III phosphoinositide‐3‐Kinase that can inhibit autophagosome formation. As shown in Figure 8A, both 3‐MA and Baicalin treatment significantly alleviated Ang II‐induced mean fluorescence intensity of MDC staining, while combination of Baicalin and 3‐MA treatment didn't further reduce the mean fluorescence intensity (compared with 3‐MA treatment alone) in Ang II stimulated H9C2 cells (Figure 8A. Interestingly, Baicalin exhibited an intriguing effect on the Ang II‐induced model, such as restoring the autophagy marker, which was evident by the up‐regulation of SQSTM1/p62 expression and the downregulation of Beclin 1 and LC3II levels. However, comparing with 3‐MA treatment alone, combination of Baicalin and 3‐MA treatment didn't further up‐regulation of SQSTM1/p62 expression and downregulation of Beclin 1 and LC3II levels in Ang II stimulated H9C2 cells (Figure 8B). In addition, the expression levels of pro‐apoptotic protein Bax, cleaved‐caspase 3 and cleaved‐caspase 9 were decreased and the level of anti‐apoptotic protein Bcl2 was increased by Baicalin or 3‐MA treatment in AngII induced H9C2 cells, indicating that autophagy is involved in Baicalin on attenuating cell apoptosis (Figure 8C). These results demonstrated that Baicalin treatment suppressed cardiomyocyte autophagy in Ang II‐induced mice and H9C2 cells.

### 3.7 Baicalin treatment suppressed AMPK/mTOR signalling pathway in vivo and in vitro

**FIGURE 9:**
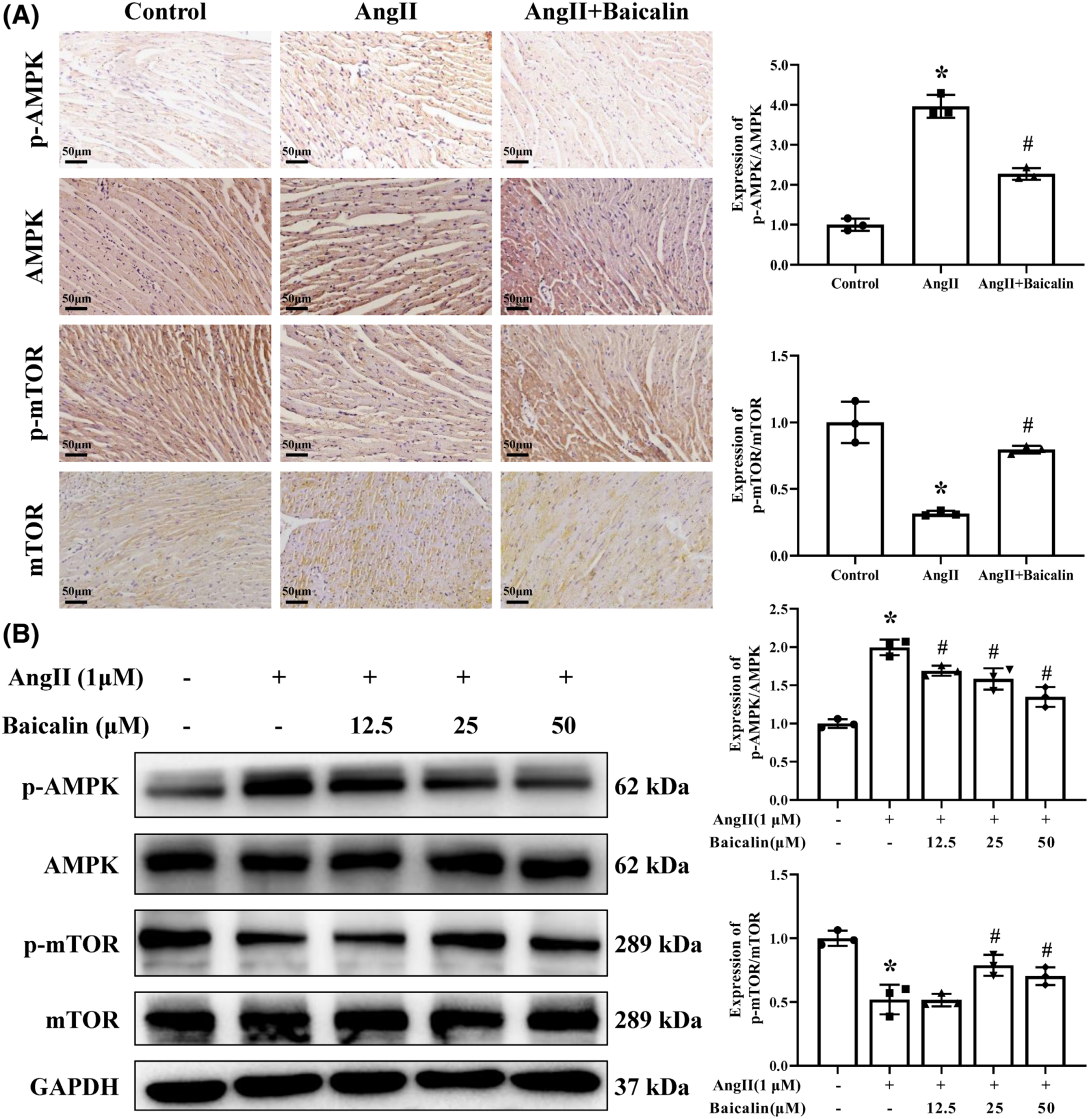
Baicalin alleviates angiotensin II (Ang II) induced hypertension‐induced heart injury via the AMPK/mTOR pathway. (A) The representative images of immunohistochemistry of p‐AMPK, AMPK, p‐mTOR and mTOR in different groups under 400× magnifications. (B) Western blotting was used to detect the expression of p‐AMPK, AMPK, p‐mTOR and mTOR in H9C2 cells. GAPDH was included as a loading control. *p < 0.05, versus Control group, ^#^ *p* < 0.05, versus Ang II group. All values are mean ± SD (*n* = 3 samples from each group).

IHC staining indicated that Ang II infusion decreased the ratio of p‐mTOR/mTOR, while increased the ratio of p‐AMPK/AMPK compared to the control group, which were all attenuated after Baicalin treatment (Figure 9A). Consistent with in vivo study, Baicalin treatment significantly increased the ratio of p‐mTOR/mTOR and decreased the ratio of p‐AMPK/AMPK in Ang II stimulated H9C2 cells (Figure 9B). These results suggested that Baicalin treatment suppressed the AMPK/mTOR signalling pathway in vivo and in vitro.

## 4 DISCUSSION

It is well known that HHD causes high fatality and unfortunately, there is currently no effective treatment available to prevent this damage.^41^, ^42^, ^43^ As reported, Baicalin could reduce the risk of worsening cardiac dysfunction.^29^ However, the effect and mechanism of Baicalin on Ang II‐induced cardiac apoptosis had not yet been reported. Therefore, the present study investigated the protective effects and the related mechanisms of Baicalin on Ang II‐induced cardiac apoptosis in vivo and in vitro. Research results showed that the effect of Baicalin on myocardial apoptosis was attributed to Ang II‐triggered excessive autophagy exogenous via the AMPK/mTOR axis.

HHD is a vital pathophysiological bases in the procession of hypertension induced heart failure and is the result of the combination of myocardial apoptosis, hypertrophy, and fibrosis.^5^, ^6^, ^19^ Chinese medicine *S. baicalensis* has been used clinically for thousands of years.^44^ Baicalin is one of the main active ingredients extracted from the Chinese herbal medicine *S. baicalensis*.^45^ Emerging evidence has established that Baicalin offers beneficial roles against the initiation and progression of cardiovascular diseases.^30^ In the present study, Baicalin treatment significantly alleviated Ang II‐induced decrease in LVEF and LVFS and increase in LVM, LV Vol;d and LV Vol;s. These results strongly indicate that Baicalin has a protective effect on Ang II‐induced cardiac injury.

A genome‐wide high‐throughput RNA‐seq analysis is helpful to completely investigate the potential molecular mechanism related to the therapeutic target and disease. In the present study, we performed an RNA‐seq analysis to identify the DETs among different groups. We identified 892 up‐regulated and 875 down‐regulated transcripts in the Ang II group. By contrast, we identified 768 up‐regulated and 823 down‐regulated transcripts in the Ang II + Baicalin group. The intersection analysis of the abovementioned DETs revealed that Baicalin treatment reversed 192 up‐regulated (top 10 transcripts: Got1, Sltm, Mier1, Tshz2, Msantd3, Ubl7, Mycbp2, Sestd1, Naa30 and Mink1) and 122 down‐regulated (top 10 transcripts: Nol8, Camsap1, Limch1, Adamtsl4, Pcdhga10, Slc4a4, Vcp, Mecp2, Flywch1 and Nap1l4) transcripts. Among them, Got1, Sltm, Sestd1, Mink1, Adamtsl4, Slc4a4, Vcp and Mecp2 have been reported to be associated with cardiovascular disease.^46^, ^47^, ^48^, ^49^, ^50^, ^51^, ^52^, ^53^ The specific functional mechanisms of these genes should be the focus of detailed research in the future. Moreover, we will further explore the specific functions of these genes, which might be important targets of Baicalin.

Target gene prediction and functional enrichment analysis are useful to clarify the potential molecular mechanism of Baicalin. The GO analysis involving the Ang II and Control groups and Ang II + Baicalin and Ang II groups revealed that Baicalin treatment could protect cells from cardiac injury due to multiple BPs (execution phase of apoptosis, autophagy, macro autophagy, ventricular cardiac muscle tissue morphogenesis), CCs (autophagosome, pre‐autophagosomal structure, AMP‐activated protein kinase complex, TOR complex, Bcl‐2 family protein complex) and MFs (death domain binding, AMP‐activated protein kinase activity). Moreover, the KEGG analysis demonstrated that there were 284 overlapping pathways in both comparisons. Among them, cardiac muscle contraction, apoptosis, autophagy, AMPK signalling pathway, mTOR signalling pathway were enriched, thereby encouraging us to further validate the underlying mechanisms.

The progressive loss of cardiomyocytes caused by cell apoptosis is one of the critical damages to HHD.^54^ Therefore, targeting cardiomyocyte apoptosis may be the primary approach to improve cardiac function and quality of life in patients with hypertension. Abundant research found that Baicalin reduced pathological changes in blood pressure and pressure overload‐induced cardiac remodelling and cardiomyocyte apoptosis.^34^, ^35^, ^36^ In this study, first, we confirmed that Baicalin treatment produced a benefit on Ang II‐induced dysfunction of the heart in vivo and in vitro. As expected, Baicalin markedly suppressed the decreased Ang II‐induced cardiomyocyte apoptosis and regulated the apoptosis‐related markers expression, including Bax, Bcl‐2, cleaved‐caspase 3 and cleaved‐caspase 9. These results implicated that Baicalin played a protective role in Ang II‐induced cardiomyocyte apoptosis.

Autophagy is a crucial and evolutionarily conserved catabolic process that plays a key role in maintaining cellular homeostasis by acting as a critical modulator for self‐protection.^10^, ^15^, ^55^ It plays an essential role in many physiological and pathological conditions by degrading damaged organelles and proteins to promote nutrient circulation and maintain homeostasis.^56^ Impaired autophagy activity is found to be closely related to the development of cardiac disease.^57^ Our current study found that Baicalin treatment directly reduced the mean fluorescence intensity of autophagic vacuoles, restored the autophagic flux, downregulated the protein expression of Beclin 1 and LC3II, while increasing the protein expression of SQSTM1/p62, which were similar with the therapeutic effects of 3‐MA (a classical autophagic inhibitor) treatment, while combination of 3‐MA treatment didn't further enhance the above therapeutic effects comparing with 3‐MA treatment alone. Interestingly, Baicalin or 3‐MA treatment down‐regulated the expression levels of pro‐apoptotic protein Bax, cleaved‐caspase 3 and cleaved‐caspase 9, while up‐regulated the level of anti‐apoptosis protein Bcl2 in AngII‐induced H9C2 cells. Thus, we can infer that Baicalin has been partly involved in mediating autophagy and apoptosis.

AMPK/mTOR signalling pathway have been characterized to regulate autophagy in response to stress and starvation.^58^, ^59^ Our results showed that Baicalin significantly increased the ratio of p‐mTOR/mTOR and decreased the ratio of p‐AMPK/AMPK in Ang II stimulated mice and H9C2 cells. These results indicated that Baicalin could inhibit the activity of the AMPK/mTOR signalling pathway, which might be the mechanism of Baicalin inhibiting cardiac apoptosis and excessive autophagy. Therefore, Baicalin inhibited Ang II‐triggered excessive autophagy, and via suppressing the activation of the AMPK/mTOR axis might be one of underlying mechanisms.

In conclusion, the present study provided convincing evidence for the first time that Baicalin suppresses cardiomyocyte apoptosis in Ang II‐induce mice and H9C2 cells through alleviating autophagy via AMPK/mTOR pathway, providing a potential novel insight for seeking therapeutic plans of HHD.

## 5 CONCLUSION

In summary, our research demonstrates that Baicalin alleviated Ang II‐induced cardiomyocyte apoptosis and autophagy, which might be related to the inhibition of the AMPK/mTOR pathway.

## AUTHOR CONTRIBUTIONS

**Ying Cheng:** Data curation (lead); methodology (lead); software (lead); writing – original draft (lead). **Mengchao Yan:** Methodology (lead); software (equal); writing – original draft (lead). **Shuyu He:** Resources (equal); software (equal). **Yi Xie:** Data curation (equal); investigation (equal). **Lihui Wei:** Resources (equal); software (equal). **Bihan Xuan:** Methodology (equal); software (equal). **Zucheng Shang:** Data curation (equal); software (equal). **Meizhu Wu:** Data curation (equal); software (equal). **Huifang Zheng:** Software (equal); supervision (equal). **Youqin Chen:** Writing – review and editing (equal). **Meng Yuan:** Writing – review and editing (equal). **Jun Peng:** Conceptualization (lead); funding acquisition (equal); methodology (equal); writing – review and editing (lead). **Aling Shen:** Conceptualization (lead); funding acquisition (equal); methodology (lead); supervision (lead).

## FUNDING INFORMATION

This study was sponsored by the National Natural Science Foundation of China (82074363 and U22A20372), the Science and Technology Major Project of Fujian Province (2019YZ014004), the Young Elite Scientists Sponsorship Program by China Association of Chinese Medicine (2021‐QNRC2‐B19), the Scientific Research Foundation for the Top Youth Talents of Fujian University of Traditional Chinese Medicine (XQB202202), the Fujian Province Natural Science Foundation, China (2021J01940) and the Development Fund of Chen Keji Integrative Medicine (CKJ2020003).

## CONFLICT OF INTEREST STATEMENT

The authors declare no conflict of interest.

## Data Availability

The data used and analysed during the current study are available from the corresponding author upon reasonable request. The RNA sequencing data used in present study are deposited at NCBI GEO number: GSE193504.
